# Laparoscopic Repair of Acute Traumatic Diaphragmatic Hernia: A Case Report

**DOI:** 10.7759/cureus.40959

**Published:** 2023-06-26

**Authors:** Rim H Charara, Rana Ibrahim, Rana Zaarour, Ali Houmani, Houssein Haidar Ahmad

**Affiliations:** 1 Internal Medicine, Lebanese University Faculty of Medicine, Beirut, LBN; 2 Research Department, Saint George Hospital, Beirut, LBN; 3 Pulmonary and Critical Care Department, Saint George Hospital, Beirut, LBN; 4 Radiology Department, Saint George Hospital, Beirut, LBN; 5 Surgery Department, Saint George Hospital, Beirut, LBN

**Keywords:** herniated abdominal organs, case study, laparoscopic repair, thoracoabdominal trauma, traumatic diaphragmatic hernia (tdh)

## Abstract

Traumatic diaphragmatic hernia (TDH) is a rare condition resulting from blunt or penetrating thoracoabdominal trauma and is characterized by the protrusion of abdominal organs into the thoracic cavity through a ruptured diaphragm. Due to its diverse clinical presentations, TDH often faces diagnostic challenges. Accurate diagnosis relies on imaging studies and surgical exploration, with surgical intervention being the primary treatment approach. This case presentation highlights a young patient who presented to Saint George Hospital following a blunt thoracoabdominal injury. The patient experienced unexplained dyspnea upon admission, and imaging revealed herniated bowels in the left hemithorax. Laparoscopic exploration confirmed a left hemi-diaphragmatic tear, with the transverse colon, omentum, most of the small bowel, and stomach herniating into the left hemithorax. The patient underwent laparoscopic repair, involving the reduction of the herniated organs into the peritoneal cavity and tension-free primary closure with gastropexy without the use of mesh for reinforcement. The patient’s postoperative course was uneventful, and complete recovery was achieved. This case report provides insights into the diagnosis and management of TDH, highlighting the importance of prompt recognition and appropriate surgical intervention in achieving successful outcomes.

## Introduction

The diaphragmatic muscle serves as a barrier between the thoracic and abdominal cavities and facilitates chest expansion during inspiration [1]. Traumatic diaphragmatic hernia (TDH) is characterized by the protrusion of abdominal hollow viscera into the thoracic cavity due to a diaphragmatic defect. TDH commonly occurs following high-energy blunt or penetrating thoracoabdominal trauma, with an incidence rate of 2.1% in blunt traumas and 3.4% between penetrating and blunt abdominal injuries [2]. TDH is a rare condition but can be life-threatening, with a mortality rate of 31% when diagnosis and repair are delayed [3]. TDH is categorized as acute or chronic, depending on the timing of the diaphragmatic rupture. Acute TDH is diagnosed immediately after traumatic injury, while chronic TDH lacks a diaphragmatic hernia diagnosis upon admission after trauma [3]. Clinical manifestations of TDH vary widely, with respiratory symptoms ranging from mild to severe, often leading to delayed diagnosis until a respiratory compromise or strangulation of herniated abdominal viscera occurs [4]. Thus, timely diagnosis and treatment are crucial to preventing serious complications. Diagnosis primarily relies on a comprehensive physical examination, complemented by imaging techniques such as a spiral computed tomography (CT) scan and endoscopic evaluation. Surgical intervention is the definitive treatment for TDH, although the approach may vary based on the case and surgeon preferences [4,5]. In this report, we present a case of acute TDH following blunt thoracoabdominal trauma successfully repaired through laparoscopic intervention involving the reduction of herniating organs in the abdominal cavity. The surgery was delayed until the patient stabilized, followed by laparoscopic exploration and correction. The patient achieved complete recovery and underwent serial imaging before discharge.

## Case presentation

**Figure 1:**
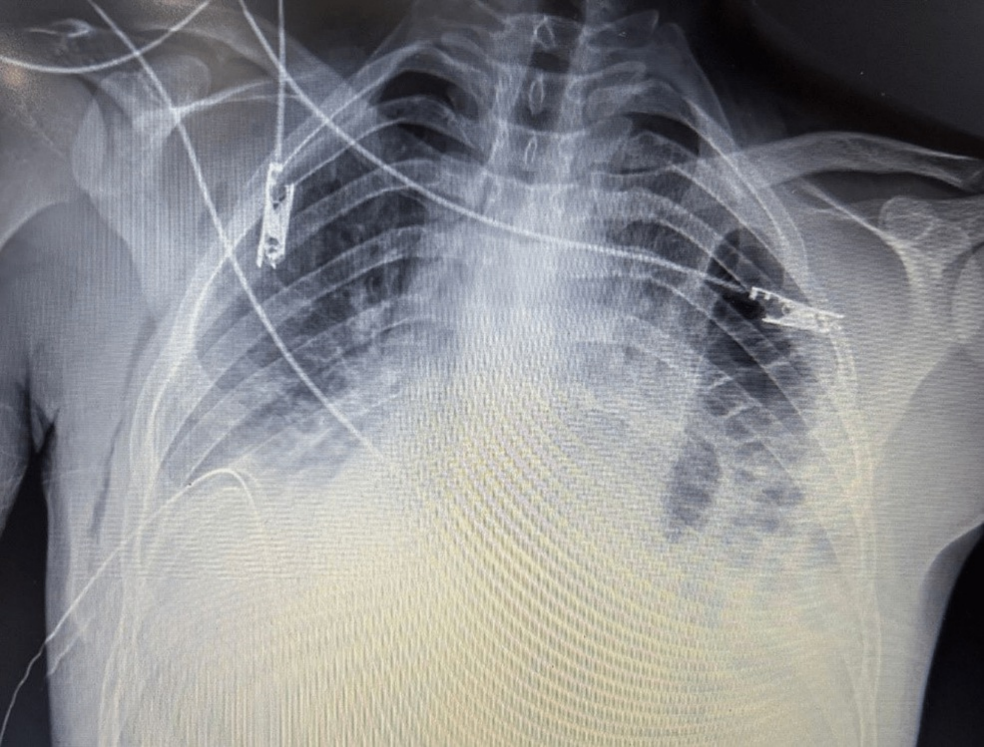
Chest X-ray showing collapsed left lung with bowel loops seen in the left hemithorax.

**Figure 2:**
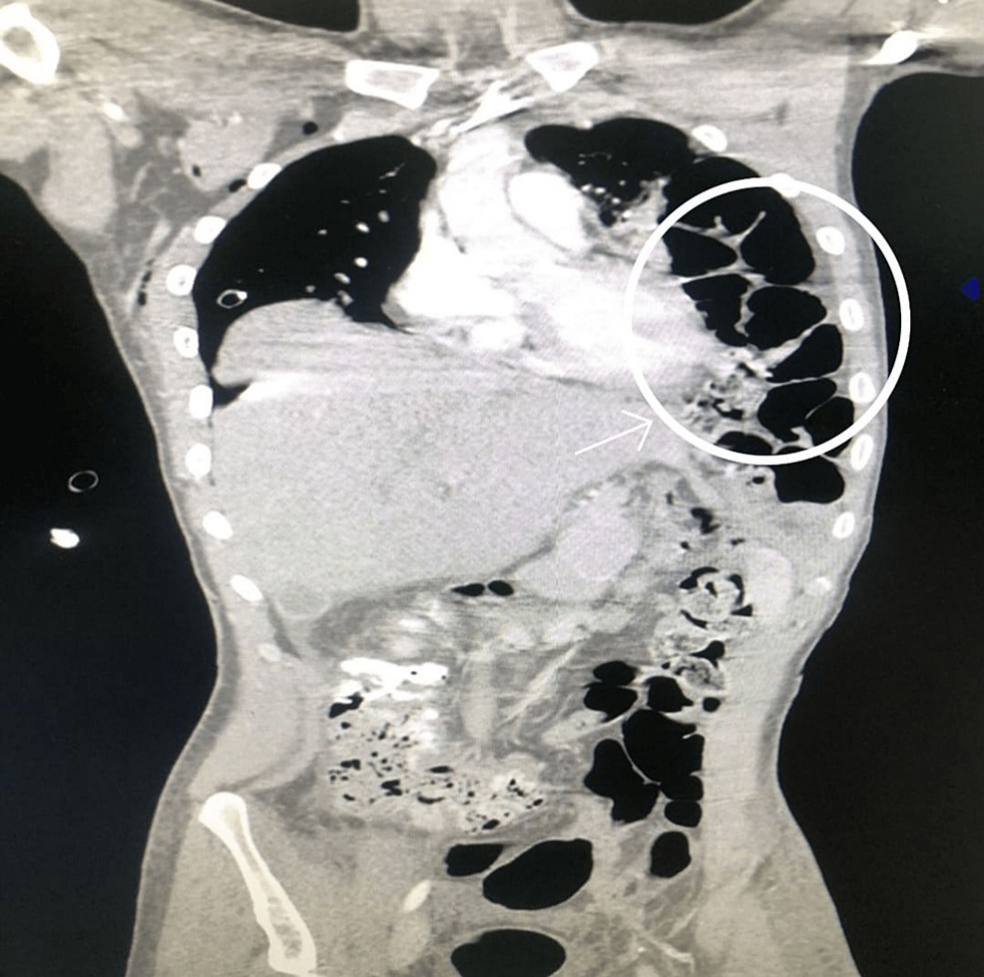
Computed tomography of the chest showing herniation of bowels in the left hemithorax.

A 20-year-old male patient was admitted to Saint George Hospital following blunt trauma to the chest, abdomen, and lower extremities. The patient had no significant past medical history. On admission, he experienced difficulty walking and complained of severe pain in his lower extremities. He was conscious, cooperative, and fully oriented, with a Glasgow Coma Scale score of 15/15. His vital signs were stable. A comprehensive physical examination was conducted, revealing a notable decrease in breath sounds on the left lung and audible bowel sounds on the left hemithorax upon auscultation. The abdomen was found to be soft and non-tender to palpation. Laboratory results were within the normal range. X-ray imaging of the lower extremities revealed a comminuted left tibial plateau fracture with subluxation of the lateral fragment, accompanied by a left ankle fracture and an undisplaced left iliac bone fracture. The chest X-ray performed upon admission showed a right hemothorax and a chest tube was inserted for several days. Serial chest X-rays and thoracoabdominal CT scans were performed, indicating a resolving hemothorax in the right lung. Additionally, parts of the small bowel and transverse colon were observed in the left thoracic cavity, along with a collapse of the left lung, suggesting a left diaphragmatic tear through which the abdominal organs were herniating (Figures 1, 2).

**Figure 3:**
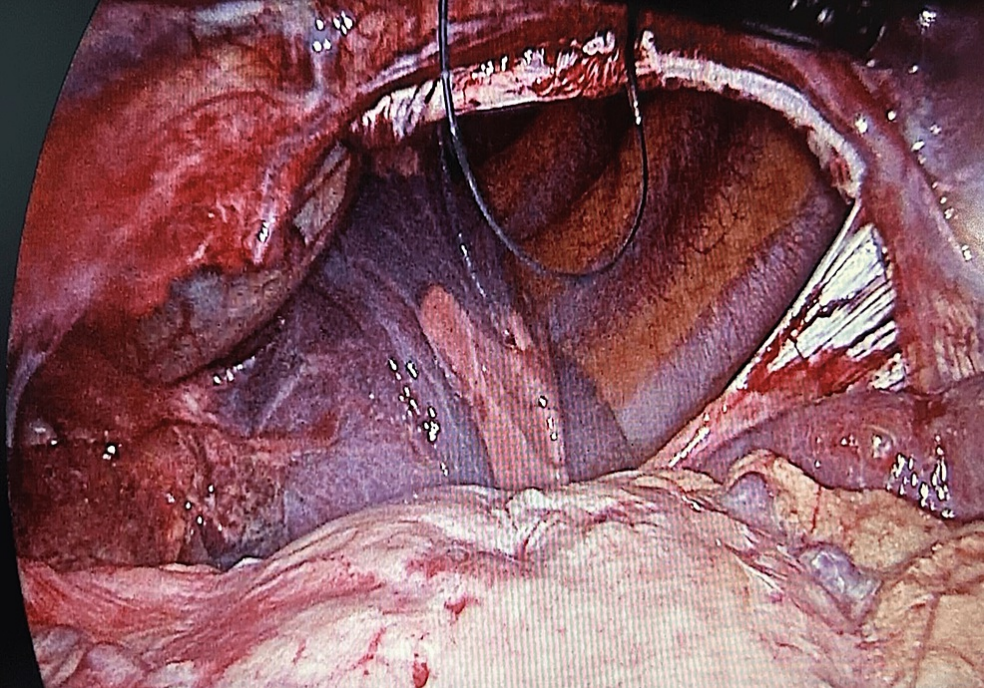
Laparoscopic image showing the diaphragmatic defect.

The patient underwent open reduction and internal fixation of the tibia and ankle by an orthopedic surgeon under spinal anesthesia, while the fracture of the iliac bone was conservatively managed. A semi-elective exploratory laparoscopy and repair were initially planned within 24 to 48 hours. However, the patient suddenly developed abdominal pain accompanied by worsening dyspnea. The onset of this new dyspnea could not be attributed to the resolving right hemothorax or underlying pneumonia. Consequently, an urgent laparoscopic intervention was performed within hours to confirm the diagnosis of left diaphragmatic rupture and repair the defect. The patient was positioned supine with open access to the abdomen, and four trocars were inserted. Exploration of the abdomen revealed a mild amount of blood in the right upper quadrant, along with a large posterolateral diaphragmatic rupture measuring more than 10 cm in length. Intrathoracic herniation of the stomach, transverse colon, omentum, and most of the small bowel was observed (Figure 3).

**Figure 4:**
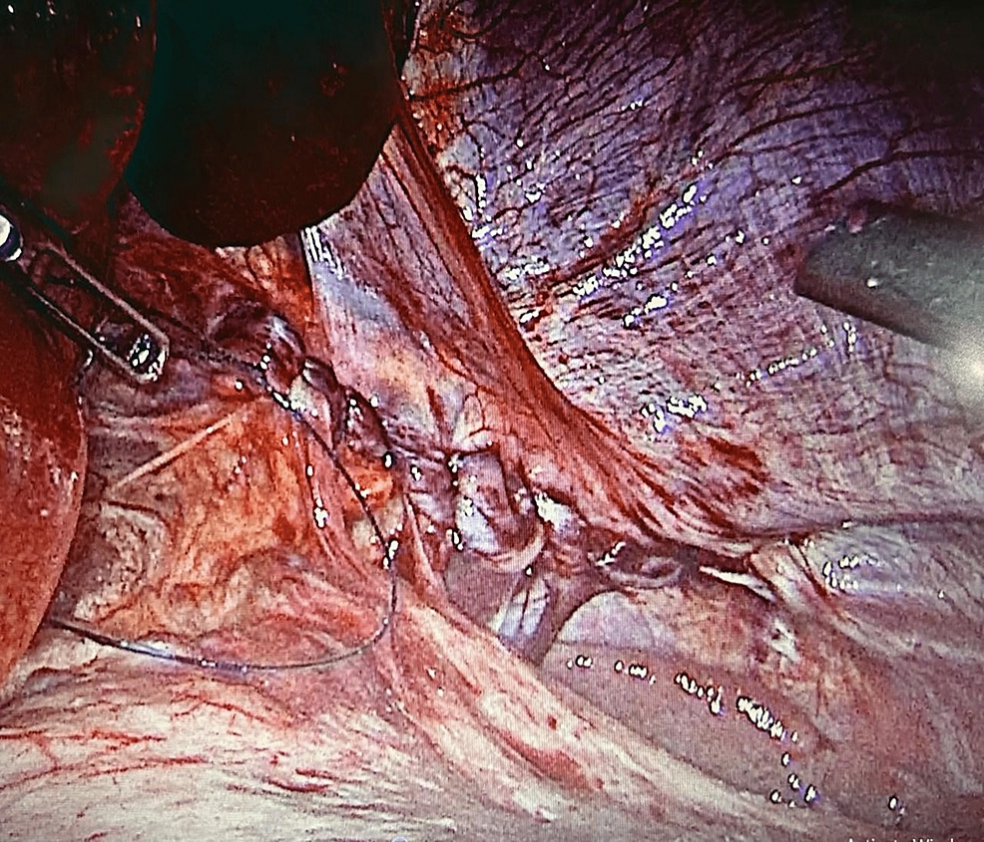
Laparoscopic image showing a tension-free primary closure of the diaphragmatic defect with slowly absorbable V-lock sutures without the use of a mesh.

**Figure 5:**
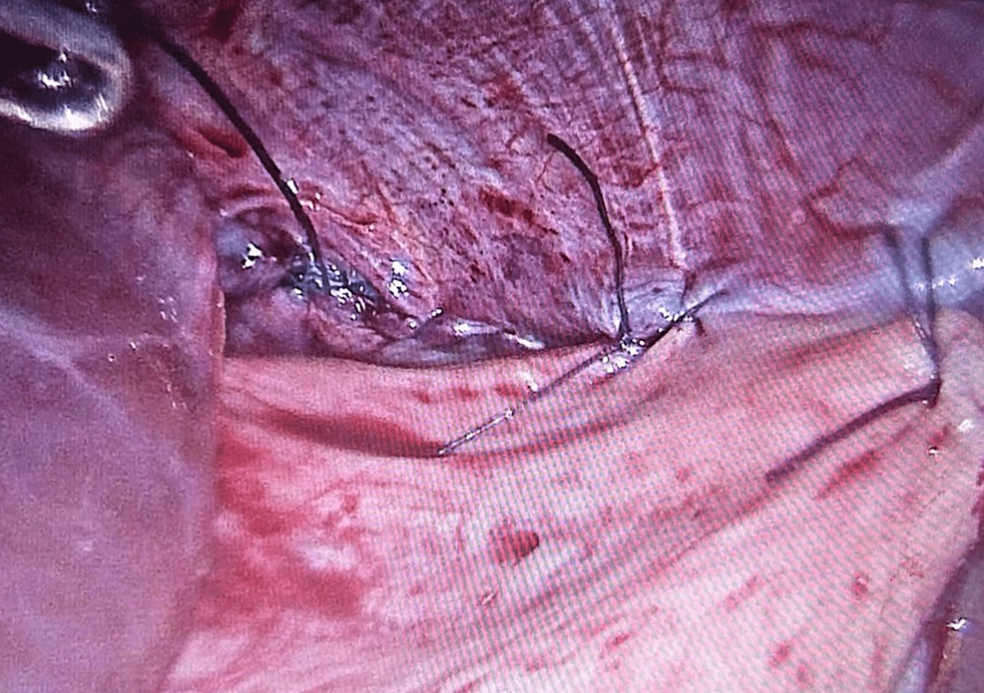
Laparoscopic image showing gastropexy where the stomach was secured to the abdominal wall.

The patient was repositioned to the reverse Trendelenburg position, and all herniated organs were gently reduced inside the peritoneal cavity. The diaphragmatic laceration was closed primarily using running 2/0 V-lock 180 sutures without tension and without the use of any prosthesis. The stomach was secured to the anterior abdominal wall using multiple interrupted sutures (Figures 4, 5).

**Figure 6:**
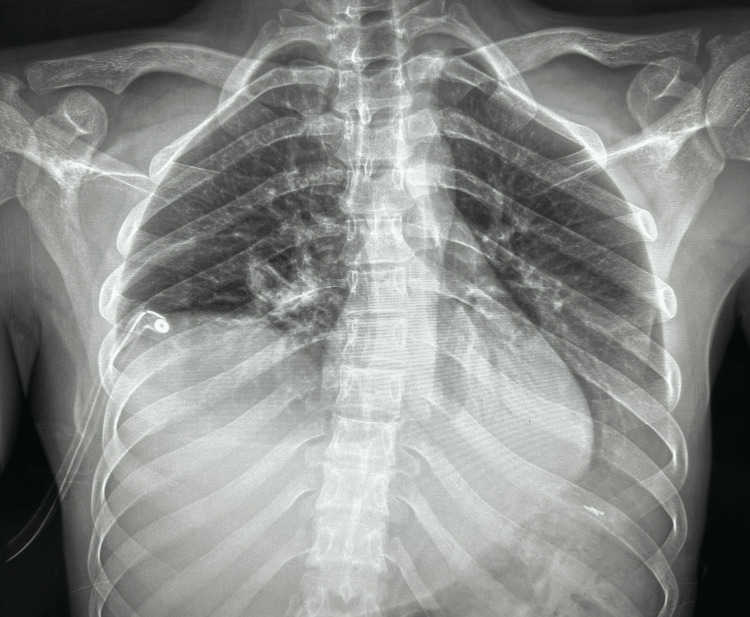
Postoperative chest X-ray after correction of the diaphragmatic defect showing a full expansion of the left lung.

A drain was placed in the left upper quadrant. During the postoperative period, the patient’s recovery was focused on chest rehabilitation, including the use of incentive spirometry and chest physiotherapy. On the second day after the operation, the patient was initiated on a clear fluid diet. A follow-up chest X-ray was performed, revealing no protrusion of abdominal organs into the chest and complete expansion of the left lung (Figure 6). The patient was discharged several days later after being closely monitored in the hospital with serial chest X-rays. A thoracoabdominal CT scan was recommended after 12 months to rule out any recurrence.

## Discussion

Laparoscopic repair has been documented in the literature as a viable approach for managing acute TDH, although the number of reported cases is limited [6,7]. Our case demonstrated the feasibility and efficacy of laparoscopic repair in acute TDH, consistent with the findings of previous studies. A similar case with acute TDH underwent laparoscopic repair [8]. The authors emphasized the advantages of laparoscopy in providing meticulous exploration of the diaphragm, reducing herniated organs, and achieving tension-free primary closure. Similarly, our patient underwent successful laparoscopic exploration, reduction of herniated organs, and primary closure without the use of mesh reinforcement.

Comparing our case to other reported cases, it is noteworthy that laparoscopic repair has emerged as a preferred approach for managing acute TDH. This minimally invasive technique offers several advantages over traditional open approaches, including reduced postoperative pain, shorter hospital stays, faster recovery, and improved cosmetic outcomes. A case series of laparoscopic repairs in TDH patients was reported, demonstrating favorable outcomes with reduced postoperative complications and mortality rates [9]. These findings further support the effectiveness of laparoscopic repair as a safe and feasible option for acute TDH. Further research involving larger patient cohorts and long-term follow-up is necessary to validate the efficacy and long-term outcomes of laparoscopic repair in acute TDH.

TDH is characterized by the displacement of abdominal contents into the chest cavity and commonly occurs as a result of blunt or penetrating injuries to the abdomen and thorax. Diaphragmatic rupture is observed in approximately 2.1% of blunt thoracoabdominal injuries and 3.4% of penetrating traumas [2,7]. Motor vehicle accidents account for around 80% of cases, but our patient’s condition was not consistent with this etiology. Other causes may include acts of violence, iatrogenic factors, or falls. Left-sided diaphragmatic hernias are more frequently encountered due to the protective barrier provided by the liver on the right side, and they are detected approximately four times more often than right-sided hernias on plain chest radiographs. Most ruptures occur on the posterolateral aspect of the diaphragm, which is relatively weaker [7].

The clinical presentation of TDH encompasses a broad spectrum, ranging from asymptomatic to moderate-to-severe respiratory compromise. Patients may present with severe chest pain, dyspnea, tachypnea, and respiratory distress. Gastrointestinal symptoms, such as abdominal pain, nausea, vomiting, a sense of fullness, and signs of gastrointestinal obstruction, may also be present. Physical examination findings may be unremarkable, except for decreased breath sounds on auscultation of the affected side and, occasionally, a scaphoid abdomen. Audible bowel sounds may be heard on the affected hemithorax [6,10]. TDHs can be classified as acute or chronic. Chronic hernias are typically associated with smaller defects, resulting in delayed diagnoses, but the size of the defect becomes larger by the time of diagnosis. Respiratory symptoms are more commonly observed in acute hernias, while gastrointestinal symptoms due to incarceration and strangulation of the small bowel and colon are more prevalent in chronic hernias [3].

Our case involved an acute diaphragmatic hernia, where the defect was diagnosed immediately following admission during the post-traumatic phase. The patient primarily presented with respiratory symptoms, confirming our earlier observations. Various imaging modalities can aid in the diagnosis of traumatic diaphragmatic hernia, including chest radiographs, ultrasounds, MRIs, and CT scans, with CT being considered the gold standard [11]. Diaphragmatic rupture does not have the ability to close spontaneously and always requires surgical intervention. The surgical approach can be via thoracotomy, laparoscopy, or laparotomy, depending on the case, available resources, expertise, and surgeon preference. In our case, exploratory laparoscopy was performed. This approach was first reported by Campos and Sipes in 1991 [12] and subsequently by Kuster et al. [13] in 1992, demonstrating its safety and efficacy for diagnosing and repairing diaphragmatic injuries.

Laparoscopically, the protruding abdominal organs can be easily and safely reduced into the abdominal cavity compared to an open-repair approach, mainly due to the expandable nature of the abdominal cavity facilitated by pneumoperitoneum. Hemodynamically stable patients with a history of previous abdominal surgeries are typically preferred candidates for laparoscopic exploration and repair. Esmer et al. have even combined thoracoscopy and laparoscopy, resulting in improved postoperative complications and reduced mortality rates [8,14]. However, laparotomy is typically reserved for hemodynamically unstable patients or those with a history of abdominal surgeries [15]. Regardless of the surgical approach, primary closure using non-absorbable sutures should be considered after reducing the abdominal organs back into the abdominal cavity [6]. In our case, slowly absorbable 2/0 V-lock 180 sutures were used instead of non-absorbable sutures.

The use of mesh for acute diaphragmatic hernias is not considered a recommended standard method, according to the American Association for the Surgery of Trauma (AAST). Mesh reinforcement is advocated when significant tissue loss occurs [10]. Several types of mesh, including composite and biological mesh for contaminated fields, can be used for reinforcement [5]. The AAST has categorized diaphragmatic injuries into five grades based on size and tissue loss. This classification ranges from superficial contusion (grade I) to lacerations less than 2 cm (grade II), lacerations between 2 cm and 10 cm (grade III), lacerations exceeding 10 cm with tissue loss less than 25 cm^2^ (grade IV), and lacerations exceeding 10 cm with tissue loss greater than 25 cm^2^ (grade V). The AAST guidelines advocate mesh reinforcement for grade VI and grade V hernias (tissue loss exceeding 20-30 cm^2^ and laceration length exceeding 10 cm) [8]. In our case, although the diaphragmatic laceration slightly exceeded 10 cm in length, it was successfully closed primarily without tension, and no mesh reinforcement was necessary.

## Conclusions

TDH is a rare condition associated with significant mortality and morbidity, particularly when the diagnosis is delayed. The diverse range of symptoms makes them challenging to identify without the aid of imaging modalities. Laparoscopic repair has emerged as the preferred approach for both diagnosing suspected hernias based on imaging findings and treating acute diaphragmatic hernias due to its demonstrated safety and efficacy. In our presented case, we successfully repaired an acute diaphragmatic hernia using a laparoscopic approach. The use of prosthesis in such cases remains a topic of debate and should be determined based on the extent of tissue loss and the size of the laceration. It is crucial to raise awareness about traumatic diaphragmatic hernias, especially in trauma centers, to facilitate early diagnosis and prevent associated complications. Further research is warranted to establish clear recommendations and guidelines for surgeons regarding the surgical approach, indications for prosthesis use, and the appropriate type of mesh for repairing acute TDHs.
